# Teriflunomide reduces relapses with sequelae and relapses leading to hospitalizations: results from the TOWER study

**DOI:** 10.1007/s00415-014-7395-7

**Published:** 2014-06-28

**Authors:** Aaron E. Miller, Richard Macdonell, Giancarlo Comi, Mark S. Freedman, Ludwig Kappos, Mathias Mäurer, Tomas P. Olsson, Jerry S. Wolinsky, Sylvie Bozzi, Catherine Dive-Pouletty, Paul W. O’Connor

**Affiliations:** 1 Icahn School of Medicine at Mount Sinai, 5 East 98th Street, Box 1138, New York, NY 10028 USA; 2 Austin Health, Heidelberg, VIC Australia; 3 Department of Neurology and Institute of Experimental Neurology, San Raffaele Scientific Institute and Vita-Salute San Raffaele University, Milan, Italy; 4 The Ottawa Hospital Research Institute, University of Ottawa, Ottawa, ON Canada; 5 University Hospital Basel, Basel, Switzerland; 6 Caritas Krankenhaus Bad Mergentheim, Bad Mergentheim, Germany; 7 Department of Clinical Neuroscience, Karolinska Institute, Stockholm, Sweden; 8 The University of Texas Health Science Center at Houston, Houston, TX USA; 9 Sanofi R&D, Chilly-Mazarin, France; 10 St Michael’s Hospital, University of Toronto, Toronto, ON Canada

**Keywords:** Clinical trial, Economics, Multiple sclerosis, Outcome assessment (healthcare), Teriflunomide

## Abstract

Teriflunomide is a once-daily oral immunomodulator approved for the treatment of relapsing–remitting multiple sclerosis. This post hoc analysis of the Phase III TOWER study evaluated the effects of teriflunomide treatment on five severe relapse outcomes: relapses with sequelae defined by an increase in Expanded Disability Status Scale (EDSS)/functional system (FS) score (sequelae-EDSS/FS) 30 days post relapse; relapses with sequelae defined by the investigator (sequelae-investigator); relapses leading to hospitalization; relapses treated with intravenous corticosteroids; and intense relapses using the definition of Panitch et al. from the EVIDENCE study based on specified increases in EDSS for severe relapses. Adjusted annualized rates for the five severe relapse outcomes were derived using a Poisson model with robust error variance, with treatment, baseline EDSS strata and region as covariates. Compared with placebo, teriflunomide significantly reduced annualized rates of relapses with sequelae-EDSS/FS [14 mg, 36.6 % (*p* = 0.0021); 7 mg, 31.3 % (*p* = 0.0104)] and sequelae-investigator [14 mg only, 53.5 % (*p* = 0.0004)], relapses leading to hospitalization [14 mg only, 33.6 % (*p* = 0.0155)], relapses requiring intravenous corticosteroids [14 mg, 35.7 % (*p* = 0.0002); 7 mg, 21.5 % (*p* = 0.0337)], and intense relapses [14 mg only, 52.5 % (*p* = 0.0015)]. Patients treated with teriflunomide 14 mg spent significantly fewer nights in hospital for relapse (*p* = 0.009) and had lower annualized rates of all hospitalizations (*p* = 0.030). Taken together, the positive effects of teriflunomide on severe relapses indicate that teriflunomide may reduce relapse-related healthcare costs.

**Electronic supplementary material:**

The online version of this article (doi:10.1007/s00415-014-7395-7) contains supplementary material, which is available to authorized users.

## Introduction

Multiple sclerosis (MS) is a chronic inflammatory autoimmune disease affecting the central nervous system [1, 2]. Relapses (or exacerbations) are one of the clinical hallmarks of MS. Relapses greatly affect the emotional and psychological state, and quality of life of patients with MS [3–6]; in some cases, relapses require patient hospitalization and are, therefore, associated with increased costs of care [5, 7].

Teriflunomide (Aubagio^®^, Genzyme, Cambridge, MA, USA) is a once-daily, oral, disease-modifying therapy approved for treatment of relapsing–remitting multiple sclerosis (RRMS) [8]. The efficacy of teriflunomide on annualized relapse rate (ARR) and disability progression in patients with relapsing forms of MS (RMS) has been assessed in two Phase III clinical trials: TEriflunomide Multiple Sclerosis Oral (TEMSO, ClinicalTrials.gov: NCT00134563) and Teriflunomide Oral in people With relapsing multiplE scleRosis (TOWER, ClinicalTrials.gov: NCT00751881) [9, 10]. In both studies, once-daily teriflunomide 14 mg significantly reduced ARR [relative reduction vs. placebo: 31.5 %, *p* = 0.0005 (TEMSO); 36.3 %, *p* = 0.0001 (TOWER)] and the risk of sustained disability progression (confirmed for 12 weeks) [hazard rate reduction vs. placebo: 29.8 %, *p* = 0.0279 (TEMSO); 31.5 %, *p* = 0.0442 (TOWER)]. Teriflunomide 7 mg also significantly reduced ARR in both studies [31.2 %, *p* = 0.0002 (TEMSO) and 22.3 %, *p* = 0.0183 (TOWER)], but had no significant effect on the risk of disability progression [9, 10]. Teriflunomide has a well-characterized safety profile, with safety results consistent across both studies and both doses; no unexpected adverse events (AEs) have been reported across the extensive teriflunomide clinical development program in long-term extension studies of up to 12 years [9–12].

A previous analysis of TEMSO showed that treatment with both doses of teriflunomide significantly reduced the risk of relapses requiring intravenous (IV) corticosteroids, relapses requiring hospitalizations at the time of initial relapse assessment, and also relapses with sequelae [confirmed by changes in Expanded Disability Status Scale (EDSS)/functional system (FS) score]. The higher dose of teriflunomide was also associated with a significant reduction in relapses with sequelae defined by the treating neurologist [13]. In addition, all hospitalizations and emergency medical facility visits were significantly reduced for patients treated with teriflunomide 14 mg [13].

This post hoc analysis of the TOWER study evaluated the consistency of the effect of teriflunomide on a range of severe relapse outcomes characterized by the same definitions used in the TEMSO analysis [13] and also assessed the intense relapses using the definition of Panitch et al. from the EVIDENCE study based on specified increases in EDSS for severe relapses [14]. The same relapse outcomes, analyzed in a pooled dataset of TEMSO and TOWER studies, are also discussed here.

## Methods

### Study designs

TOWER was a Phase III, multinational, multicenter, randomized, double-blind, placebo-controlled, parallel-group study designed to assess the efficacy and safety of teriflunomide in patients with RMS. Detailed methodology for this study has been published previously and is only briefly described here [10]. TOWER enrolled ambulatory patients with RMS, aged 18–55 years, with EDSS scores ≤5.5 and ≥1 relapse in the previous 12 months or ≥2 relapses in the prior 24 months. Patients were randomized 1:1:1 to either teriflunomide 14 mg, teriflunomide 7 mg or placebo, once daily.

Treatment duration in TOWER was variable and ended 48 weeks after the last patient was randomized into the study. Patients were excluded from TOWER if they experienced a relapse within 30 days prior to randomization. The primary objective of the study was to assess the efficacy of teriflunomide in reducing ARR.

### Standard protocol approvals, registrations and patient consent

TOWER was conducted in accordance with the International Conference on Harmonisation Guidelines for Good Clinical Practice and the Declaration of Helsinki. The protocol was approved by central and local ethics committees and each site’s institutional review board, and patients gave written consent prior to the study.

### Study evaluations

Post hoc analyses assessed the effects of teriflunomide treatment on outcomes from protocol-defined confirmed relapses (see below) occurring between treatment randomization and treatment discontinuation. Relapses were defined as the appearance of a new clinical sign/symptom or clinical worsening of a previous sign/symptom, which persisted for a minimum of 24 h in the absence of fever. In case of suspected relapse, patients were instructed to contact the investigator and to be examined within 7 days to confirm the occurrence of the relapse. Relapses were confirmed by the treating neurologist and required either a 1-point increase in at least two FS functions, or a 2-point increase in at least one FS function (excluding bowel/bladder and cerebral), or an increase of ≥0.5 points in EDSS score (or ≥1 point when EDSS = 0) from the previous clinically stable assessment. Suspected and confirmed relapses could be treated with IV corticosteroids according to the judgment of the investigator; the preferred standardized treatment was IV methylprednisolone sodium succinate 1 g administered once daily for 3–5 days.

The following severe relapse outcomes were evaluated: relapses with sequelae, which are neurological consequences post relapse, and relapses with severe intensity at onset.

Sequelae were classified as either those associated with confirmed changes in EDSS/FS (sequelae-EDSS/FS) or those recorded by the investigator (sequelae-investigator). Sequelae-EDSS/FS corresponded to an increase in EDSS/FS persisting for at least 30 days after the onset of the relapse and followed the same definition that was used previously for a confirmed relapse. As sequelae can persist beyond 30 days, increases in EDSS/FS scores were also assessed at 60, 90, 120, 150, and 180 days post relapse. When two relapses occurred successively without an intervening EDSS/FS assessment, the first relapse was excluded from the analysis. Sequelae-investigator were defined as relapses with incomplete neurological recovery at the end of the relapse, and were recorded by the treating neurologist on the relapse section of the Case Report Form; relapses were documented as either recovering with sequelae (worsened intensity, ongoing or unknown) or without sequelae, and the end of a relapse was assessed by the investigator.

Relapses with severe intensity at onset were assessed in two ways: as relapses requiring healthcare resources (i.e., confirmed relapses leading to hospitalization or those requiring IV corticosteroids) or as intense relapses that met the definition of a ‘severe relapse’ from the EVIDENCE study of Panitch et al. [14]; intense relapses were defined as those that exceeded criteria for a moderate relapse (i.e., an EDSS score increase of 1 or 2 points, a 2-point FS change in one or two systems, or 1-point change in four or more systems at the time of confirmed relapse assessment [14]).

Finally, the impact of teriflunomide on overall healthcare resource consumption was assessed by documenting the number of nights spent in hospital for a confirmed relapse and all hospitalizations. Only the nights spent in hospital for relapses between the randomization date and the last treatment administration were included. All hospitalization relates to any hospitalizations recorded on the AE form or relapse form.

### Statistical analysis

All analyses were performed on the modified intention-to-treat population, defined as all patients randomized and exposed to study medication for at least 1 day (*N* = 1,165), and analyzed in the treatment group according to randomization. All analyses on relapse outcomes were performed on protocol-defined confirmed relapses. For each severe relapse outcome, the number and percentage of patients without relapses were calculated. Annualized rates were estimated for each severe relapse, and also for the number of nights spent in hospital and all hospitalizations. Annualized rates were adjusted using a Poisson regression model with robust error variance, as for the primary outcome of the study (ARR). The total number of the outcome of interest was defined as the response variable; treatment, EDSS strata at baseline and region were covariates; log-transformed standardized treatment duration was defined as an offset variable. A *χ* ^2^ test was used to compare percentages of relapses leading to hospitalization among relapses requiring IV corticosteroid.

## Results

### Baseline demographics

The baseline demographics and disease characteristics of the TOWER study population have been published elsewhere [10]. Populations were well balanced with no significant differences observed among the three treatment groups.

### Effect of teriflunomide treatment on severe relapses

#### Relapses with sequelae: sequelae-EDSS/FS

**Fig. 1:**
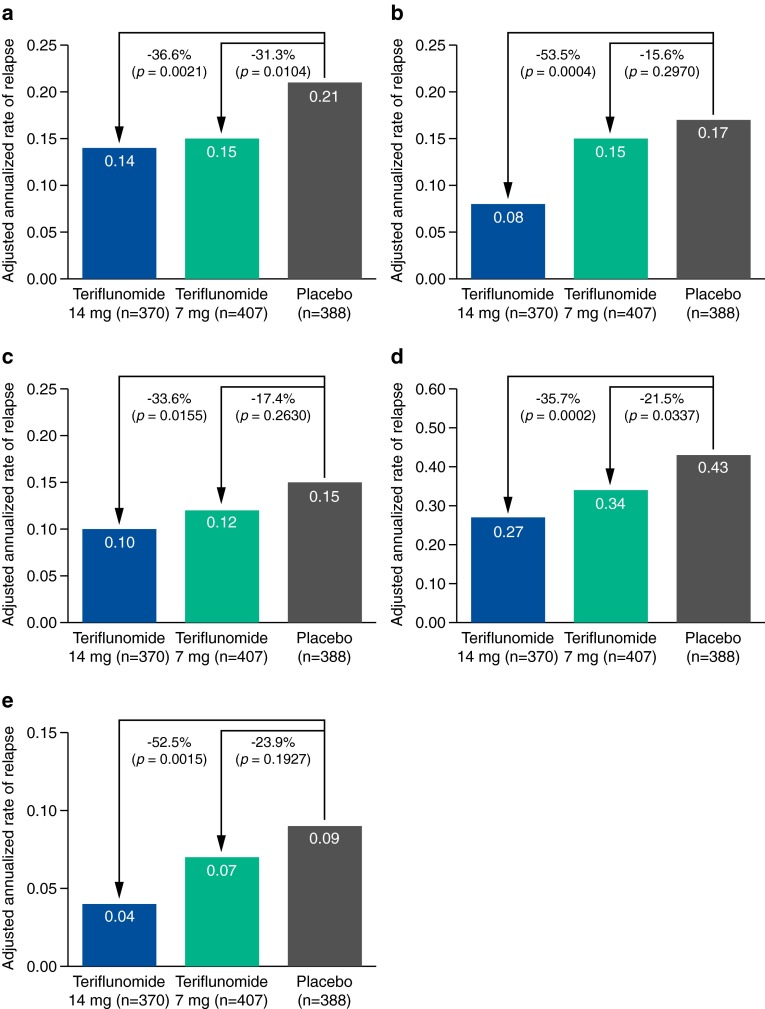
Adjusted annualized rates for each relapse outcome analyzed in TOWER: **a** relapses with sequelae-EDSS/FS; **b** relapses with sequelae-investigator; **c** relapses leading to hospitalization; **d** relapses requiring IV corticosteroids; **e** intense relapses (intense relapses using the definition of Panitch et al. from the EVIDENCE study, based on specified increases in EDSS for severe relapses [14]). *EDSS* Expanded Disability Status Scale, *FS* functional system, *IV* intravenous

**Fig. 2:**
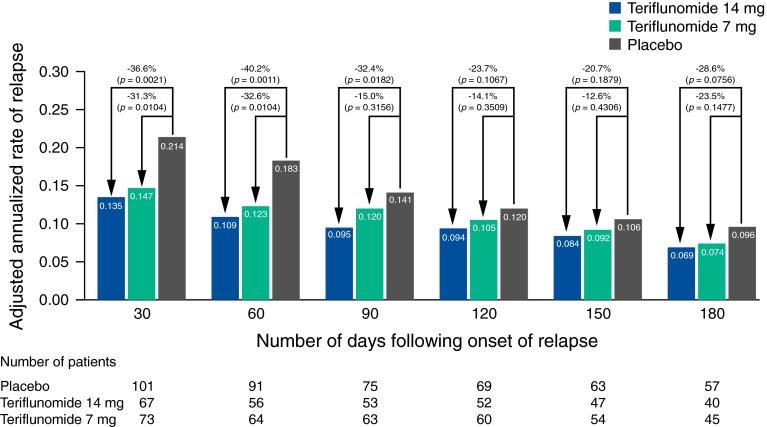
Adjusted annualized rate of relapse with sequelae-EDSS/FS over time in TOWER. *EDSS* Expanded Disability Status Scale, *FS* functional system

The annualized rate of relapse with sequelae-EDSS/FS at least 30 days after the start of relapse was lower in both teriflunomide groups than in the placebo group (teriflunomide 14 mg, 0.14; teriflunomide 7 mg, 0.15; placebo, 0.21) (Fig. 1a). Treatment with teriflunomide 14 and 7 mg significantly reduced the annualized rate of relapse with sequelae-EDSS/FS at least 30 days after start of relapse by 36.6 % (*p* = 0.0021) and 31.3 % (*p* = 0.0104), respectively, compared with placebo. When compared with placebo, the significant treatment effect on relapses with sequelae-EDSS/FS was also observed at 60 and 90 days post relapse with teriflunomide 14 mg, and at 60 days post relapse with teriflunomide 7 mg (Fig. 2).

#### Relapses with sequelae: sequelae-investigator

The annualized rate of relapse with sequelae-investigator was numerically lower in both teriflunomide groups than in the placebo group (teriflunomide 14 mg, 0.08; teriflunomide 7 mg, 0.15; placebo, 0.17) (Fig. 1b). Teriflunomide 14 mg significantly reduced the annualized rate of relapse with sequelae-investigator by 53.5 % (*p* = 0.0004 vs. placebo), while the reduction observed with teriflunomide 7 mg was not significant (*p* = 0.297 vs. placebo).

#### Relapses leading to hospitalization

The annualized rate of relapses leading to hospitalization was lower in both teriflunomide groups compared with placebo (teriflunomide 14 mg, 0.10; teriflunomide 7 mg, 0.12; placebo, 0.15) (Fig. 1c). Teriflunomide 14 mg significantly reduced the annualized rate of relapse leading to hospitalization by 33.6 % (*p* = 0.0155 vs. placebo), while the 17.4 % reduction observed with teriflunomide 7 mg vs. placebo was not significant (*p* = 0.2630) (Fig. 1c).

#### Relapse requiring IV corticosteroids

The annualized rate of relapse requiring IV corticosteroids was lower in both teriflunomide groups than for placebo (teriflunomide 14 mg, 0.27; teriflunomide 7 mg, 0.34; placebo, 0.43) (Fig. 1d). Both doses of teriflunomide significantly reduced the annualized rate of relapse requiring IV corticosteroids (teriflunomide 14 mg, 35.7 %, *p* = 0.0002; teriflunomide 7 mg, 21.5 %, *p* = 0.0337) compared with placebo.

Since in some countries patients are hospitalized for steroid infusions, we have distinguished the proportion of relapses requiring IV corticosteroids from the proportion of relapses leading to hospitalization, by calculating the proportion of relapses requiring IV corticosteroids without hospitalization. Most of the relapses requiring IV corticosteroids did not lead to hospitalization (teriflunomide 14 mg, 54 %; teriflunomide 7 mg, 51 %; and placebo, 54 %), with no significant differences across the treatment groups (teriflunomide 14 mg vs. placebo, *p* = 0.9719; teriflunomide 7 mg vs. placebo, *p* = 0.6325).

#### Intense relapses

The annualized rate of intense relapse was numerically lower in both teriflunomide groups than in the placebo group (teriflunomide 14 mg, 0.04; teriflunomide 7 mg, 0.07; placebo, 0.09) (Fig. 1e). Teriflunomide 14 mg significantly reduced the annualized rate of severe relapse by 52.5 % compared with placebo (*p* = 0.0015). Although teriflunomide 7 mg reduced the annualized rate of severe relapse by 23.9 %, this reduction was not significant (*p* = 0.1927 vs. placebo).

### Effect of teriflunomide treatment on healthcare resource consumption

The annualized number of nights spent in hospital per patient for relapses was significantly reduced by 46 % for teriflunomide 14 mg (*p* = 0.0086 vs. placebo) (Table 1). For 1,000 patients treated with teriflunomide 14 mg, this would translate into 696 nights saved per year, when compared with placebo. Teriflunomide 7 mg was associated with a non-significant reduction of 27 % (*p* = 0.2816 vs. placebo).

**Table 1:** Adjusted annualized number of nights spent in hospital for relapse per patient and adjusted annualized rate of all hospitalization for relapse or serious AE (TOWER modified-ITT population)

	Teriflunomide 14 mg (*n* = 370)	Teriflunomide 7 mg (*n* = 407)	Placebo (*n* = 388)
Adjusted annualized number of nights spent in hospital for relapse per patient^a^			
Estimate (95 % CI)	0.81 (0.55, 1.21)	1.10 (0.61, 1.99)	1.51 (1.00, 2.27)
Relative risk vs. placebo (95 % CI)	0.54 (0.34, 0.86)	0.73 (0.41, 1.30)	
*p* value vs. placebo	0.0086	0.2816	–
Adjusted annualized rate of all hospitalization for relapse or serious AE^b^			
Adjusted annualized rate (95 % CI)	0.21 (0.17, 0.27)	0.25 (0.20, 0.31)	0.29 (0.23, 0.37)
Relative risk vs. placebo (95 % CI)	0.72 (0.54, 0.97)	0.86 (0.64, 1.15)	–
*p* value vs. placebo	0.0299	0.3145	–

*AE* adverse event, *CI* confidence interval, *ITT* intention to treat

^a^Derived using Poisson regression model with robust error variance with total number of nights of hospitalization for relapse as response variable; treatment, Expanded Disability Status Scale strata at baseline and region as covariates; and log-transformed patient years as an offset variable

^b^Derived using Poisson regression model with robust error variance with total number of nights spent in hospital (for relapse or AE) as response variable; treatment, Expanded Disability Status Scale strata at baseline and region as covariates; and log-transformed patient years as an offset variable

The annualized rate of all hospitalizations (due to a relapse or an AE) was lower in both teriflunomide groups than in the placebo group (teriflunomide 14 mg, 0.21; teriflunomide 7 mg, 0.25; placebo, 0.29) (Table 1). Teriflunomide 14 mg significantly reduced the annualized rate of all hospitalizations by 27.8 % (*p* = 0.0299 vs. placebo), while the 14 % relative reduction observed with teriflunomide 7 mg was not significant (*p* = 0.3145 vs. placebo) (Table 1).

## Discussion

This analysis of TOWER demonstrated that teriflunomide 14 mg significantly reduced the occurrence of relapses with sequelae (based either on EDSS/FS score changes or by investigator assessment), relapses leading to hospitalization, and relapses treated with IV corticosteroids. These results are consistent with those observed in a similar analysis of the Phase III TEMSO study [13]. Thus, in conclusion, teriflunomide has demonstrated consistent and beneficial outcomes on relapses with sequelae and relapses requiring healthcare resources compared with placebo across two Phase III clinical trials. This observation also reinforces the consistency of effects of teriflunomide treatment reported for both clinical and safety outcomes in the TEMSO and TOWER studies [9, 10]. In addition, this analysis extends the results observed in TEMSO by showing a beneficial effect of teriflunomide 14 mg on annualized rates of intense relapses.

Similarities in the study designs, timing, and nature of evaluations and patient populations in the TEMSO and TOWER trials allowed for pooling of data from both studies to further assess relapse outcomes in a larger study population [9, 10]. Consistent with observations in TOWER and TEMSO [13], respectively, teriflunomide significantly reduced occurrence of severe relapses, including relapses with sequelae and relapses requiring healthcare resources vs. placebo (Supplementary Table 1). In addition, the annualized rate of all hospitalizations was significantly reduced with teriflunomide 14 mg but not with teriflunomide 7 mg vs. placebo (Supplementary Table 2). The analysis of the pooled data from TEMSO and TOWER provides an estimate of overall treatment effect on relapse outcomes in a larger dataset and further supports the beneficial effect of teriflunomide on severe relapses––those with sequelae, those requiring healthcare resources, and those that can be described as intense.

Reducing the frequency and severity of relapses is an important goal of MS treatment. Patients with MS who experience relapses face a variety of challenges, including restrictions in daily activities, social involvement, role within the family and ability to continue working [3, 4]. An analysis of the impact of relapses on a range of health-related quality of life (HRQoL) and fatigue outcomes in the pooled TEMSO and TOWER cohort (regardless of treatment allocation) demonstrated that HRQoL and fatigue were adversely affected in patients experiencing relapses, with a more profound impact from severe relapses (using the same definitions applied in this analysis) [15]. Treatments that have a beneficial effect on severe relapses are, therefore, important in the management of MS.

Patients treated with teriflunomide 14 mg in TOWER had a lower rate of hospitalization and spent fewer nights in hospital because of relapse compared with patients randomized to placebo. These results are consistent with those seen in the TEMSO study [13] and supplement the limited number of studies associating disease-modifying therapies with fewer hospitalizations [16–19]. Limiting the number of nights spent in hospital is a challenge. Over the past decade, annual admissions due to MS increased by 40 % in the USA [20]. The costs associated with hospitalization are also significant, with relapse-related hospitalization approximately six times higher than the overall cost of managing patients with a relapse in an outpatient setting [21]. Therefore, the observed reductions in the rates of relapse-related and all hospitalizations associated with teriflunomide treatment may have a potential impact on the substantial direct and indirect healthcare expenditures associated with the disease [7]. The management of relapse, which includes hospitalization for corticosteroid infusion and/or for relapses, varies across countries and such analyses should be interpreted with caution. Additionally, this analysis was performed post hoc and as such was not powered a priori to evaluate the outcomes analyzed here. Further prospective analyses could be conducted to confirm these conclusions. However, the results presented here are strongly supported by the fact that TOWER enrolled patients from different countries with different approaches to relapse management, and with a similar distribution across the three treatment groups.

In conclusion, this post hoc analysis of TOWER replicates and extends the results obtained in TEMSO, and shows that teriflunomide use was associated with a reduction in severe relapses, including those associated with neurological sequelae and the consumption of healthcare resources. These findings indicate that teriflunomide may reduce relapse-related healthcare costs and further support the use of teriflunomide in patients with RMS.

## Electronic supplementary material

Below is the link to the electronic supplementary material.

Supplementary material 1 (DOC 44 kb)
