# Supplementary material for: Teriflunomide reduces relapses with sequelae and relapses leading to hospitalizations: results from the TOWER study
Source: J Neurol. 2014 Jun 28;261(9):1781–8. doi: 10.1007/s00415-014-7395-7 (PMC4155167; doi:10.1007/s00415-014-7395-7)
Supplement: Supplementary file 1 — Supplementary material 1 (DOC 44 kb) [file 415_2014_7395_MOESM1_ESM.doc]

**SUPPLEMENTARY MATERIALS**

**Supplementary Table 1** Adjusted annualized rates and relapse rate reduction associated with teriflunomide treatment (pooled TEMSO and TOWER modified-ITT population)

|  | Adjusted annualized rates | | | Relapse rate reduction (%)^a^ | |  |
| --- | --- | --- | --- | --- | --- | --- |
|  | Teriflunomide  14 mg  (*n* = 728) | Teriflunomide  7 mg  (*n* = 772) | Placebo  (*n* = 751) | 14 mg vs. placebo | 7 mg vs. placebo |  |
| Relapses with sequelae-EDSS/FS^b^ | 0.16 | 0.18 | 0.26 | 36.4  *p* < 0.0001 | 31.4  *p* < 0.0001 |  |
| Relapses with sequelae-investigator^c^ | 0.09 | 0.15 | 0.19 | 53.1  *p* < 0.0001 | 20.4  *p* = 0.0456 |  |
| Relapses leading to hospitalization | 0.09 | 0.12 | 0.16 | 45.5  *p* < 0.0001 | 25.5  *p* = 0.0195 |  |
| Relapses requiring IV corticosteroids | 0.28 | 0.32 | 0.43 | 34.5  *p* < 0.0001 | 25.4  *p* = 0.0002 |  |
| Intense relapse^d^ | 0.048 | 0.066 | 0.088 | 45.0  *p* = 0.0002 | 24.6  *p* = 0.0596 |  |

^a^Derived using a Poisson regression model with robust error variance with treatment, Expanded Disability Status Scale strata at baseline, region and study as covariates, and log-transformed patient years as an offset variable

^b^Incomplete neurological recovery, defined by an increase in the Expanded Disability Status Scale (EDSS) or functional system (FS) score 30 days post relapse

^c^Incomplete neurological recovery, as assessed by the investigator at the end of the relapse

^d^Intense relapses using the definition of Panitch et al. from the EVIDENCE study, based on specified increases in EDSS for severe relapses [14]

*ITT* intention-to-treat, *IV* intravenous

**Supplementary Table 2** Adjusted annualized number of nights spent in hospital for relapse per patient and adjusted annualized rate of all hospitalization for relapse or serious AE (pooled TEMSO and TOWER modified-ITT populations)

|  | Teriflunomide  14 mg  (*n* = 728) | Teriflunomide  7 mg  (*n* = 772) | Placebo  (*n* = 751) |
| --- | --- | --- | --- |
| Adjusted annualized number of nights spent in  hospital for relapse per patient^a^ |  |  |  |
| Estimate (95% CI) | 0.71 (0.52, 0.95) | 0.91 (0.60, 1.38) | 1.39 (1.04, 1.86) |
| Relative risk vs. placebo (95% CI) | 0.51 (0.36, 0.72) | 0.65 (0.43, 0.99) |  |
| *p* value vs. placebo | 0.0001 | 0.0455 | - |
| Adjusted annualized rate of all hospitalization for  relapse or serious AE^b^ |  |  |  |
| Adjusted annualized rate (95% CI) | 0.19 (0.16, 0.23) | 0.23 (0.19, 0.27) | 0.27 (0.23, 0.32) |
| Relative risk vs. placebo (95% CI) | 0.70 (0.56, 0.86) | 0.84 (0.68, 1.03) | - |
| *p* value vs. placebo | 0.0009 | 0.0988 | - |

^a^Derived using Poisson regression model with robust error variance with total number of nights of hospitalization for relapse as response variable; study, treatment, Expanded Disability Status Scale strata at baseline and region as covariates; and log-transformed patient years as an offset variable

^b^Derived using Poisson regression model with robust error variance with total number of nights spent in hospital (for relapse or AE) as response variable; treatment, Expanded Disability Status Scale strata at baseline, region and study as covariates; and log-transformed patient years as an offset variable

*AE* adverse event, *CI* confidence interval, *ITT* intention to treat
